# A case-control study of Dietary Approaches to Stop Hypertension (DASH) diets, colorectal cancer and adenomas among Iranian population

**DOI:** 10.1186/s12885-021-08786-5

**Published:** 2021-09-25

**Authors:** Saeede Jafari Nasab, Matin Ghanavati, Pegah Rafiee, Alireza Bahrami, Nazanin Majidi, Cain C. T. Clark, Amir Sadeghi, Mohammad Houshyari, Ehsan Hejazi

**Affiliations:** 1 Student Research Committee, School of Nutrition and Food Science, Isfahan University of Medical Sciences, Isfahan, Iran; 2 Department of Clinical Nutrition and Dietetics, Faculty of Nutrition Sciences and Food Technology, National Nutrition and Food Technology, Research Institute, Shahid Beheshti University of Medical Sciences, Tehran, Iran; 3 Department of Nutrition, Islamic Azad University, Science and Research Branch, Tehran, Iran; 4 Centre for Intelligent Healthcare, Coventry University, Coventry, CV15FB UK; 5 Gastroenterology and Liver Diseases Research Center, Research Institute for Gastroenterology and Liver Diseases, Shahid Beheshti University of Medical Sciences, Tehran, Iran; 6 Department of Radiation Oncology, Faculty of Medicine, Shahid Beheshti University of Medical Sciences, Tehran, Iran

**Keywords:** Colorectal cancer, Colorectal adenomas, Adenomatous polyps, Dietary approaches to stop hypertension, DASH score, Dietary indexes, Diet quality indexes

## Abstract

**Background and aims:**

Colorectal cancer (CRC) is the third most common cancer, worldwide. Recently, much attention has been given to the association between Dietary Approaches to Stop Hypertension (DASH) and CRC, however, data on colorectal adenomas (CRAs) as its precursor are scarce. Thus, the purpose of this case-control study was to investigate the association of DASH score with the risk of CRC and CRA in Iranian adults.

**Method:**

A total of 499 participants, including 129 CRC and 130 CRA cases, along with 240 controls, were asked about their dietary intake via a validated questionnaire. The DASH score was then calculated based on a priori methods and categorized in quartiles. Multivariate logistic regression was performed to assess the association of DASH score and the risk of CRC and CRA.

**Results:**

After adjusting for confounding variables, adherence to the DASH diet was associated with a reduction in the risk of CRC and CRA, respectively (OR of 4th versus 1st quartile = 0.04, 95% CI: 0.01–0.11, OR = 0.10, 95% CI: 0.04–0.22). Also, subgroup analysis based on gender showed that women and men with a higher DASH score had a significantly lower risk of CRC and CRAs.

**Conclusion:**

The results of this study demonstrated that adherence to a DASH dietary pattern could reduce the risk of CRC and CRA in men and women. Promoting a DASH eating plan can be helpful in reducing the risk of CRC.

## Background

Colorectal cancer (CRC) is the third most common cancer worldwide, and was recently estimated to be responsible for 694,000 deaths globally [1]. The distribution of CRC incidence varies by country, with the highest rates in well developed countries [2]. However, recent lifestyle changes in less developed countries have elicited a rapid rise of CRC incidence in these countries. Indeed, CRC is the fourth most common cancer in Iran and its’ incidence has been increasing during the past decade [3].

Inflammatory bowel disease, age, smoking, physical activity, obesity, and diet are well known risk factors of CRC [4, 5]. Also, the vast majority of CRCs arise from colorectal adenomas (CRAs); these are benign lesions that take approximately 10 years to become cancerous and people with history of adenomas are at an increased risk of CRC [6].

Among modifiable risk factors of CRC, diet has gained much attention [7]; indeed, with the goal of reducing disease risk and preventing chronic disease, including cancers, several dietary recommendations and patterns have been advocated [8, 9]. Moreover, various scoring systems, such as Healthy Eating Index, Dietary Approaches to Stop Hypertension (DASH), and Mediterranean Style Dietary Pattern Score have been used to evaluate the adherence of populations to those dietary recommendations [10–12].

DASH is a dietary pattern, rich in fruits, vegetables, nuts, seeds, whole grain cereals, moderate in low-fat dairies, and low in sodium and added sugars. It was initially developed for management of high blood pressure [13, 14]; yet, in addition to blood pressure, some studies have shown an association between the DASH dietary pattern and lower incidence of other chronic disease, such as cancers [15, 16]. Although several, individual, components of DASH dietary pattern are associated with a reduced risk of CRC (whole grains, dairy product and lower amount of red meat, sodium and extra sugar) and CRA (red meat and extra carbohydrate), this association has not been affirmed for all components of this dietary pattern [17–21]. Accordingly, rather than concentrating on single foods and nutrients, utilization of the whole DASH plan in investigating diet-CRA- CRC relations can be beneficial [14]. Indeed, some studies have reported an inverse association between adherence to the DASH pattern and CRC [22, 23], and two studies have shown this association with CRA [24, 25].

Previous studies have principally focused on CRC, however, data pertaining to CRA are sparse. Also, to the best of our knowledge, data about the relationship between DASH pattern adherence and CRC and CRA are predominantly confined to western countries, with scant evidence from less developed countries, such as Iran. Therefore, the objective of this study was to determine whether DASH dietary pattern adherence is associated with CRC and CRA, as a precursor of CRC, in a case-control study of Iranian adults.

## Method

### Study design and population

This hospital-based case-control study was conducted in 3 major referral hospital (Taleghani, Shohadae Tajrish and Emam Hosein). The study participants had already been recruited for another study [26]. Briefly, CRC and CRA cases were patients with colonoscopy and histologically confirmed CRC and CRA, aged 30–79, with no history of cancers, polyps and inflammatory bowel diseases (IBD), and diagnosed in the preceding 3 months before the interview. Over the same period of time, controls were assigned randomly from patients admitted to the same hospitals for a wide spectrum of diseases other than cancers and polyps and IBD and did not follow any special diet. Controls were frequency-matched with cases by sex and age (10-year groups) and they were aged 30-79y. After exclusion of subjects with incomplete food frequency questionnaire (FFQ) and implausible energy intake estimates (outside the range of ±3 standard deviation from the mean), 499 participants (240 controls, 129 CRC and 130 CRAs) were included in the analysis. Informed consent was provided by all of the participants. Study protocol was approved by the ethics Committee of Shahid Beheshti University of Medical Sciences.

### Dietary assessment

In this study, usual dietary intake of participants one year before diagnosis of diseases in cases, and one year prior to interview in controls was assessed through a valid and reliable semi-quantitative food frequency questionnaire, consisting of 148 foods and beverages [27]. For each item, participant were asked to specify how often (daily, weekly and monthly) they consumed foods based on a standard serving size. Then reported intakes were converted to weight equivalent (i.e. g, mg) per day. Energy and nutrients intake were estimated using United States Department of Agriculture (USDA) food composition table. However, for some traditional Iranian foods, such as traditional bread, the Iranian food composition table was used [28].

### Calculation of DASH score

The method introduced by Fung [29] was used to measure dietary DASH score. Initially, to undo the effect of individual energy intake on the components of the DASH score, each food group intake was calculated per 1000 kcal. Then, participants were classified into quintiles and energy adjusted intake of 8 food groups and nutrients including fruits, vegetables, nuts and seeds and legumes, low-fat dairy products, whole grains which are targeted in the DASH score (adequacy components) and sodium, sweetened beverages, and red and processed meat which are minimized in DASH score (moderation components), were calculated. Individuals in highest quintiles of 5 adequacy components received 5 points and those in the lowest quintiles received minimum score of 1. Regarding the intakes of moderation components, participants in the lower quintiles of intakes scored higher points (i.e., the lowest quintiles are assigned 5 points and the highest quintiles, 1 point). Finally. The total DASH score (ranged from 8 to 40) was provided by summing up the scores of 8 components for each individual.

### Covariates’ assessment

In addition to dietary assessment, necessary information about sociodemographic, characteristics, family history of CRC and other cancers, smoking habit, medical history (comorbidities, medications, and vitamin/mineral supplements intake), and usual cooking techniques were asked in a face-to-face interview. Height and weight were measured to the closest 0.1 cm and 100 g, respectively. Furthermore, physical activity was assessed by a validated questionnaire [30].

### Statistical analysis

SPSS version 21.0 (SPSS Inc., Chicago, IL, 113 USA) was used to conduct Statistical analyses. Data were analyzed for normality using the Kolmogorov-Smirnov test, and are showed as means ± SD (for normally distributed data) or median and interquartile range (for non-normally distributed data). The differences between participants across the quartiles of energy-adjusted DASH score were compared using analysis of variance (ANOVA) or Kruskal-Wallis test (Kruskal-Wallis test used for non-normally distributed data) and the chi-square tests for quantitative variables and qualitative variables, respectively. Dietary DASH score was categorized into 4 quartiles based on the distribution among controls. Binary logistic regression was applied to estimate odds ratios (ORs) and 95% confidence intervals for quartiles of DASH score. BMI (kg/m2), age (year), alcohol consumption, physical activity (MET), and history of diabetes, Coronary heart disease (CHD) and hypertension, family history of cancer were considered as potential confounders and were included in the models. The *p*-value of < 0.05 was considered statistically significant and all reported *p*-values are two-tailed.

## Results

Table 1 demonstrates the main characteristic of cases and controls. There was no statistically significant difference among participants (cancers and controls, adenomas and controls) in relation to BMI, family history of colorectal cancer in first-degree relatives, smoking status, vitamin D supplement, multivitamin use and energy intake. However, family history of cancer in first-degree relatives of CRC cases was higher than controls (51.2% versus 32.9%; *p*-value< 0.01). Compared to controls, having at least one comorbidity such as diabetes was seen more in adenoma cases (36.9% versus 15.8%; *p*-value< 0.01) and the calcium supplement consumption was higher in adenoma cases than controls (24.6% versus 14.6; *p*-value = 0.01). Besides, the median value for physical activity was significantly higher in controls than adenomas [39.02 (36.51–41.33) vs. 37.75(34.67–40.40); *p*-value< 0.01]. Controls had lower intake of salt compared to CRC and CRA cases (p-value = 0.002). Common ways of cooking among controls differed significantly from cancer and adenomas patients, (p-value< 0.05). In details, cancer cases consumed more grilled and fried foods compare to controls and steamed food was consumed lower in adenomas than in controls.

**Table 1:** The main characteristics of the cases and controls

Variables	Controls (*n* = 240)	Cancers (*n* = 129)	Adenomas (*n* = 130)	*P*-value^£^	*P*-value^†^
Age (years)	56 (50–61.75)	59 (49.25–64)	58 (51–64)	‡	‡
Gender (male)	133 (55.4)	66 (51.2)	59 (45.4)	‡	‡
BMI (kg/m2)	26.53 (24.10–29.40)	25.70 (23.08–29.74)	26.79 (23.86–29.40)	0.23	0.95
Smoking (yes)	42 (17.5)	26 (20.2)	27 (20.8)	0.53	0.11
Comorbidities (yes) ^¥^	38 (15.8)	17 (13.2)	48 (36.9)	0.84	0.002^**^
Diabetes (yes)	20 (8.3)	12 (9.3)	12 (9.2)	0.75	0.77
Hypertension (yes)	13 (5.4)	4 (3.1)	23 (17.7)	0.34	0.01^**^
Coronary Heart diseases (yes)	5 (2.1)	1 (0.8)	13 (10.0)	0.31	0.00^**^
Family history of cancer in first degree (yes)	89 (32.9)	66 (51.2)	48 (36.9)	0.001^**^	0.43
Colorectal cancer family history in first degree relatives (yes)	18 (7.5)	10 (7.8)	17 (13.1)	0.15	0.08
Common ways of cooking food					
Fried	55 (22.9)	40 (31)	18 (13.8)	0.001^**^	0.06
Boiled	81 (33.8)	41 (31.8)	34 (26.2)	0.75	0.82
Grilled	5 (2.1)	0 (0)	4 (3.1)	0.02^**^	0.52
Steam cook	3 (1.3)	2 (1.6)	0 (0)	0.32	0.006^**^
Combined	96 (40)	46 (35.7)	74 (56.9)	0.001^**^	0.06
Level of salt intake					
Low	127 (52.9)	44 (34.1)	44 (34.1)	0.002^**^	0.002^**^
Normal	79 (32.9)	64 (49.6)	64 (49.6)	0.08	0.08
High	34 (14.2)	21 (16.3)	21 (16.3)	0.07	0.07
Physical activity (MET/h/day)	39.02 (36.51–41.33)	39.30 (37.17–40.90)	37.75 (34.67–40.40)	0.85	0.003^*^
Monthly intake of 50,000 IU Vitamin D supplement (yes)	56 (23.3)	28 (21.7)	40 (30.8)	0.72	0.11
Daily intake of 500 mg Calcium supplement (yes)	35 (14.6)	28 (21.7)	32 (24.6)	0.08	0.01^**^
Multivitamin use every day (yes)	18 (7.5)	16 (12.4)	9 (7)	0.12	0.84
Energy intake (kJ/day)	9549.06 (8122.15–11,140.99)	9154.93 (7714.92–11,087.06)	9221.62 (7648.02–11,608.63)	0.35	0.35

^‡^Matched variables of the study, ^£^*p*-value between cancers and controls, ^**†**^*p*-value between adenomas and controls

Values are presented as median (Q1-Q3) for quantitative variables or n (%) for qualitative variables

* Significant difference (Mann-Whitney U, *p*-value < 0.05) ** Significant difference (Chi-Square, *p*-value < 0.05)

¥ Comorbidities are defined as diabetes, hypertension and coronary heart disease

*MET* Metabolic equivalent

Table 2 shows the distribution of participants in DASH score quartiles, according to main characteristics. Participants in the highest DASH quartile had lower sodium intake (*p* < 0.001) and were older (58.19 vs 54.29y, *p* = 0.002) compared to the lowest quartile.

**Table 2:** The main characteristics of the participants according to quartiles of energy-adjusted DASH score

Variables	DASH score quartiles				*P*-value^*^
	Quartile 1	Quartile 2	Quartile 3	Quartile 4	
	< 20	21–24	25–28	> 29	
Age (years)^‡^	54.29(11.76)	54.19(9.72)	57.19(9.44)	58.19(8.56)	0.002
Gender ^‡^					
Woman	58(41.7)	66(50.4)	62(51.2)	55(50.9)	0.34
Man	81(58.3)	65(49.6)	59(48.8)	53(49.1)	
BMI (kg/m2)	26.71(4.04)	26.91(5.21)	26.61(4.36)	27.04(3.69)	0.87
Energy intake (kcal)	2341.1(678.5)	2357.1(681.8)	2299.1(659.7)	2299.7(552.5)	0.85
Physical activity (MET/day)	36.82(7.78)	38.62(14.85)	39.86(11.75)	40.24(9.8)	0.07
Education levels					
Illiterate	20(14.6)	20(15.5)	17(14)	6(5.6)	0.21
Low	99(72.3)	91(70.5)	87(71.9)	80(74.1)	
High	18(13.1)	18(14)	17(14)	22(20.4)	
Smoking (yes)	35(25.2)	21(16)	21(17.4)	18(16.7)	0.32
Comorbidities (yes) ^¥^	27(19.4)	24(18.3)	31(25.6)	20(18.5)	0.44
Diabetes (yes)	10(7.2)	10(7.6)	13(10.7)	11(10.2)	0.68
Hypertension (yes)	12(8.6)	9(6.9)	13(10.7)	6(5.6)	0.49
Coronary Heart diseases (yes)	5(3.6)	3(2.3)	7(5.8)	4(3.7)	0.54
Family history of cancer in first degree (yes)	62(44.6)	50(38.2)	37(30.6)	44(40.7)	0.13
Colorectal cancer family history in first degree relatives (yes)	13(9.4)	12(9.2)	9(7.4)	11(10.2)	0.87
Level of salt intake					
Low	40(28.8)	58(44.3)	62(51.2)	69(63.9)	
Normal	70(50.4)	51(38.9)	49(40.5)	32(29.6)	0.00
High	29(20.9)	22(16.8)	10(8.6)	7(6.5)	
Alcohol consumption	11(7.9)	4(3.1)	4(3.3)	4(3.7)	0.11
Daily intake of multi vitamins and minerals (yes)	8(5.8)	15(11.5)	7(5.8)	13(12)	0.13

Values are mean (SD) or number (percentage)

^‡^Matched variables of the study, ^*^p-value between quartiles of DASH score

Independent sample t-test was used for continuous variables and Chi-square was used for categorical variables. MET: Metabolic equivalent

¥ Comorbidities are defined as diabetes, hypertension and coronary heart disease

Energy-adjusted DASH score and DASH score components are shown in Table 3. Compared to control group, patients with CRC and CRAs had significantly lower DASH score (21, 22 vs 27, *p* < 0.001). Patients in both CRA and CRC groups ate less servings of vegetables, fruits, whole grains and nuts, legumes, and seeds than the control group (*p* < 0.01). The consumption of sodium, red and processed meats, and sweetened beverages were significantly higher in CRC and CRA groups compared to the control group.

**Table 3:** A comparison between the case (Cancer and adenomas) and control groups based on daily intake of the energy-adjusted DASH score components

DASH score components	control	Colorectal cancer	Adenomas	*p*-value*
	(*n* = 240)	(*n* = 129)	(*n* = 130)	
Energy-adjusted DASH score	27(23–30)	21(19–24)	22(19.7–26)	< 0.001
Vegetables (serving/1000 kcal)	1.67(0.84)	1.20(0.49)	1.46(0.82)	< 0.001
Fruits (serving/1000 kcal)	1.12(0.50)	0.79(0.32)	0.98(0.45)	< 0.001
Whole grains (serving/1000 kcal)	1.43(1.16)	1.09(0.81)	1.27(1.05)	0.01
Nuts, legumes and seeds (serving/1000 kcal)	0.55(0.38)	0.47(0.32)	0.50(0.38)	0.09
Low fat dairy (serving/1000 kcal)	0.73(0.44)	0.75(0.47)	0.75(0.44)	0.91
Red and processed meats (serving/1000 kcal)	0.29(0.20)	0.59(0.39)	0.47(0.30)	< 0.001
Sweetened beverages (serving/1000 kcal)	0.06(0.12)	0.08(0.12)	0.10(0.15)	0.01
Sodium (g/d)	1857.1(512.5)	2155.69(579.1)	2149.2(587.6)	< 0.001

Values are mean (SD) or median (IQR)

* *P*-value estimated using ANOVA or Kruskal-wllis test

Crude and multi-variable adjusted ORs and 95% confidence interval for risk of CRC and CRA, based on quartile of energy-adjusted DASH score, are provided in Table 4. We found that patients in highest quartile of DASH score had a significant reduction in the risk of both CRC and CRA vs. the lowest quartile. After adjusting for confounding variables, including BMI (kg/m^2^), age (year), alcohol consumption, physical activity (MET), history of diabetes, CHD and hypertension and family history of cancer, adherence to the DASH diet was associated with a reduction in the risk of CRC and CRA, respectively (OR = 0.04, 95% CI: 0.01–0.11, OR = 0.10, 95% CI: 0.04–0.22). Also, subgroup analysis based on gender showed that both women and men with higher DASH scores had significantly lower risk of CRC and CRAs.

**Table 4:** OR and 95% confidence interval for risk of colorectal cancer, adenoma and colorectal cancer plus adenoma based on quartile of energy-adjusted DASH score

Dietary DASH score	Quartile1 < 20	Quartile2 21–24	Quartile3 25–28	Quartile4 > 29	P for trend
Colorectal cancer					
Women					
Case (control)	19(14)	23(23)	16(28)	5(42)	
Crude OR (95% CI)	1.00	0.73(0.29–1.81)	0.42(0.16–1.06)	0.08(0.02–0.27)	< 0.001
Adjusted OR* (95% CI)	1.00	0.76(0.29–1.97)	0.42(0.16–1.11)	0.07(0.02–0.24)	< 0.001
Adjusted OR**(95% CI)	1.00	0.89(0.31–2.54)	0.42(0.14–1.24)	0.07(0.02–0.26)	< 0.001
Men					
Case (control)	37(22)	22(26)	5(40)	2(45)	
Crude OR (95% CI)	1.00	0.50(0.23–1.09)	0.07(0.02–0.21)	0.02(0.00–0.12)	< 0.001
Adjusted OR* (95% CI)	1.00	0.47(0.21–1.06)	0.06(0.02–0.18)	0.02(0.00–0.10)	< 0.001
Adjusted OR**(95% CI)	1.00	0.63(0.26–1.48)	0.07(0.02–0.24)	0.02(0.00–0.13)	< 0.001
Pooled					
Case (control)	56(36)	45(49)	21(68)	7(87)	
Crude OR (95% CI)	1.00	0.59(0.33–1.05)	0.19 (0.10–0.37)	0.05(0.02–0.12)	< 0.001
Adjusted OR* (95% CI)	1.00	0.57(0.31–1.04)	0.17(0.09–0.34)	0.04 (0.01–0.10)	< 0.001
Adjusted OR**(95% CI)	1.00	0.69(0.36–1.31)	0.22(0.11–0.44)	0.04(0.01–0.11)	< 0.001
Adenoma					
Women					
Case (control)	25(14)	20(23)	18(28)	8(42)	
Crude OR (95% CI)	1.00	0.58(0.28–1.21)	0.38(0.18–0.79)	0.13(0.05–0.32)	< 0.001
Adjusted OR* (95% CI)	1.00	0.57(0.27–1.19)	0.32 (0.15–0.68)	0.04(0.04–0.27)	< 0.001
Adjusted OR**(95% CI)	1.00	0.42(0.16–1.12)	0.25(0.09–0.69)	0.06 (0.02–0.20)	< 0.001
Men					
Case (control)	22(22)	17(26)	14(40)	6(45)	
Crude OR (95% CI)	1.00	0.56(0.26–1.22)	0.33(0.15–0.73)	0.11(0.04–0.30)	< 0.001
Adjusted OR* (95% CI)	1.00	0.65(0.27–1.53)	0.35(0.15–0.81)	0.13(0.04–0.37)	< 0.001
Adjusted OR**(95% CI)	1.00	0.68(0.26–1.72)	0.30 (0.11–0.80)	0.14(0.04–0.44)	< 0.001
Pooled					
Case (control)	47(36)	37(49)	32(68)	14(87)	
Crude OR (95% CI)	1.00	0.57(0.37–1.06)	0.36(0.19–0.66)	0.12 (0.06–0.25)	< 0.001
Adjusted OR* (95% CI)	1.00	0.57(0.31–1.07)	0.30 (0.16–0.57)	0.10(0.04–0.21)	< 0.001
Adjusted OR**(95% CI)	1.00	0.60(0.31–1.15)	0.31 (0.16–0.61)	0.10(0.04–0.22)	< 0.001
Colorectal cancer plus Adenoma					
Women					
Case (control)	44(14)	43(23)	34(28)	13(42)	< 0.001
Crude OR (95% CI)	1.00	0.59(0.27–1.30)	0.38(0.17–0.84)	0.09(0.04–0.23)	< 0.001
Adjusted OR* (95% CI)	1.00	0.55(0.24–1.24)	0.34(0.15–0.77)	0.08(0.03–0.20)	< 0.001
Adjusted OR**(95% CI)	1.00	0.59(0.25–1.39)	0.35(0.15–0.83)	0.07 (0.02–0.18)	
Men					
Case (control)	59(22)	39(26)	19(40)	8(45)	< 0.001
Crude OR (95% CI)	1.00	0.55(0.27–1.12)	0.17(0.08–0.36)	0.06(0.02–0.16)	0.03
Adjusted OR* (95% CI)	1.00	0.55(0.27–1.12)	0.15(0.07–0.33)	0.05(0.02–0.14)	< 0.001
Adjusted OR**(95% CI)	1.00	0.64(0.30–1.35)	0.16 (0.07–0.37)	0.06(0.02–0.16)	
Pooled					
Case (control)	103(36)	82(49)	53(68)	21(87)	< 0.001
Crude OR (95% CI)	1.00	0.58(0.34–0.98)	0.27(0.16–0.45)	0.08 (0.04–0.15)	< 0.001
Adjusted OR* (95% CI)	1.00	0.56(0.33–0.95)	0.24 (0.14–0.41)	0.07(0.03–0.13)	< 0.001
Adjusted OR**(95% CI)	1.00	0.64(0.37–1.10)	0.26 (0.10–0.47)	0.07(0.03–0.14)	

*Model 1: Adjusted for age (year)

**Model 2: Adjusted for BMI (kg/m^2^), age (year), alcohol consumption, physical activity (MET), history of diabetes, CHD and hypertension, family history of cancer

## Discussion

In this case-control study of Iranian men and women, we observed that a higher DASH score was associated with a reduced risk of CRC and CRAs in Iranian adults. In addition, this association was seen in subgroup analysis based on gender, where a higher DASH score was related to lower risk of both CRC and CRA in men and women.

The findings of our study are in line with some other studies that displayed an inverse relationship between DASH score and the risk of CRC [22, 31] and CRAs [24, 25]. Indeed, considering studies that performed sex-specific analysis, four studies showed a significant inverse association between higher DASH scores and CRC risk only in men [31–33] and one study only in women [34]. However, another study reported this relationship was present only in women, where the authors noted a reduced risk of CRC in higher quartiles of DASH score [35]. Indeed, disparities between studies could be due to the following reasons: differences in etiology of CRC and responses to diet in men and women, hormone replacement therapy (HRT) and oral contraceptive (OCP) consumption in women which are independently and inversely associated to CRC risk [33, 36] and considering colon and rectum cancer separately or as a single cancer in analyses. Our study agrees with the result of a newly published systematic review and meta-analysis which reported an inverse significant association between higher DASH scores and reduced risks of CRC in both genders [21]. However, risk reduction in our study is far different from previous studies. This difference could represent a random variation, favored by the small sample size of some studies, or could be due to adjustment for different confounders, using self-administered FFQs in some of studies which are prone to self-report bias, whereas we administered face-to face interview by trained interviewers.

The DASH dietary pattern emphasizes the consumption of plant foods. Phytochemical and other antioxidants in these foods exert their anti-tumorigenic characteristics via inhibition of CRC cell growth (suppression of NF-κB pathway), inducing apoptosis, and reaction with reactive oxygen species [37, 38]. Further, they can interact with CRA and CRC cell growth by blocking the natural cell cycle [39]. Fiber content of plant foods is known to reduce the risk of CRC and CRA by increasing fecal volume, decreasing transit time, and therefore, decreasing the interaction of carcinogen with colonic epithelium, production of short chain fatty acids, and insulin resistance [40, 41]. The DASH dietary pattern is also low in red and processed meat; indeed, the high fat content of red meat, and heterocyclic amines and polycyclic aromatic hydrocarbons produced from cocking meat at high temperatures, in addition to nitrosyl-heme molecules in processed meats that result in N-nitroso compounds formation, are associated with increased risk of CRC and CRA [42, 43]. The DASH diet also advocates lower intakes of sugar-sweetened beverages; although the relationship between such beverages and CRC and CRA has not been fully confirmed, hyperinsulinemia and increased insulin growth factor-1 (IGF-1) consequential to consumption of these drinks can effect on carcinogenesis and conversion of adenomas to malignancies through crucial pathways, such as mitogen-activated protein kinase (MAPK) pathway and phosphoinositide 3-kinase (PI3K) pathway [44–46]. An important feature of the DASH diet is low sodium intake; accordingly, results of studies on the association of sodium and higher risk of CRC are not particularly strong, but some studies have reported positive associations between sodium and CRC risk [47]. Indeed, considering the synergic interaction between components of the DASH dietary pattern and CRC and CRA, rather than individual components, can be helpful in providing public messages to reduce the risk of CRC, and CRA as a precursor of CRC.

Although this study presents an important and novel addition to the literature, there are some limitations that should be considered. First, recall and selection bias cannot be avoided in case-control studies; however, we sought to minimize such biases by recruiting incident cases along with hospital controls. Second, we did not have information about HRT and OCP use in women, so the results could be affected by them, and they should be considered as potentially confounding variables in future work. Another limitation of our study is that although sample size is not low, when we classified participants according to various factors, the number of people decreased in each quartile. Concomitant to the aforementioned limitations, our study has some strengths that are noteworthy. First, to our knowledge, this is the first study on the association of DASH diet score and the risk of CRC and CRA in Iran, and in the Middle East. Second, we used Fung’s method to calculate DASH score, which includes wider ranges of possible scores (1 through 5), while some scores, such as Dixon’s, have only two values for components (0 for not meeting and 1 for meeting recommendations) [23]. Third, we used a validated FFQ, administered by trained nutritionists, to more accurately estimate the main exposure [27].

## Conclusion

In conclusion, the results of this study demonstrated that adherence to a DASH dietary pattern could reduce the risk of colorectal cancer and colorectal adenomas in men and women. Moreover, promoting a DASH eating plan, as a balanced and healthy diet, in dietary guidelines could be helpful in reducing the risk of CRC in less-developed countries.

## Data Availability

The datasets used and/or analysed during the current study are available from the corresponding and first author on reasonable request.

## Abbreviations

CRC: Colorectal cancer
CRAs: Colorectal adenomas
DASH: Dietary Approaches to Stop Hypertension
IBD: Inflammatory bowel diseases
USDA: United States Department of Agriculture
ANOVA: Analysis of variance
MET: Physical activity
CHD: Coronary heart disease
ORs: Odds ratios
HRT: Hormone replacement therapy
OCP: Oral contraceptive
IGF-1: Insulin growth factor-1
MAPK: Mitogen-activated protein kinase
PI3K: Phosphoinositide 3-kinase.
